# Psychomotor Abilities of Professional Handball Players

**DOI:** 10.3390/ijerph16111909

**Published:** 2019-05-30

**Authors:** Krzysztof Przednowek, Maciej Śliż, Justyna Lenik, Bartosz Dziadek, Stanisław Cieszkowski, Paweł Lenik, Dorota Kopeć, Katarzyna Wardak, Karolina H. Przednowek

**Affiliations:** 1 Faculty of Physical Education, University of Rzeszow, 35-959 Rzeszow, Poland; msliz@ur.edu.pl (M.Ś.); ju_lenik@ur.edu.pl (J.L.); bdziadek@ur.edu.pl (B.D.); scieszkowski@ur.edu.pl (S.C.); pw_lenik@ur.edu.pl (P.L.); dkopec@ur.edu.pl (D.K.); karprzed@ur.edu.pl (K.H.P.); 2 Faculty of Medicine, University of Rzeszow, 35-959 Rzeszow, Poland; wardakk@ur.edu.pl

**Keywords:** team games, professional players, psychomotor computer tests, movement time, reaction time

## Abstract

The main purpose of the paper was to evaluate selected psychomotor abilities of handball players depending on the competition class (league), position on the court, training seniority and the dominant hand. The study covered a group of 40 handball players (age: 24.02 ± 3.99), while 50 non-training men (age: 22.90 ± 1.13) formed the control group. Studies were performed using Test2Drive computer tests. The following four tests were used for measuring psychomotor fitness: simple reaction time test, choice reaction time test, hand-eye coordination test and spatial anticipation test. An analysis revealed that handball players had better reaction times and movement times than the control group. The league, position on the court, training seniority and the dominant upper limb were analysed for their impact on the reaction time and movement time in handball players. An analysis of psychomotor abilities of handball players with regard to the league revealed that in the majority of tests the Superliga players had a shorter reaction time than players in lower leagues.

## 1. Introduction

Handball is an Olympic discipline game where successful performance depends on a number of basic abilities in particular strength, power, speed and endurance. Creativity in combination with speed and strength as well as coordination makes this sport very attractive but tough to play [1]. Statistical data about the handball game from tournaments at the Olympic Games, World Championships and European Championships from the last years indicates that the effectiveness of the game in a quick attack were improved and the element of quick start of the game after losing a goal in handball is used in 80% by European teams. Moreover the active playing time of the attack has also changed from 37 min to 42 min and the number of attacks increased from 45 in the 1960s to almost 70 in the 21st century. It shows that that contemporary handball has definitely become a more dynamic and faster team game than it used to be [2,3,4].

Evaluation of motor skills and anthropometric characteristics of handball players was a subject matter of a number of studies. Jensen et al. (1997) [5] showed a significant effect of the intertwined strength and speed training on speed of movement. Similar research was made by Hoff et al. (1995) [6] who observed an improvement in the speed of the throw. Influence of the strength training used during the sports season was analysed by Gorostiaga et al. (2006) [7] which contributed to a significant improvement in the maximum upper body strength and the speed of ball throw, while no changes were noted in the strength of lower limbs. The impact of experience (sports seniority) on effectiveness of the game was also analysed [8,9,10]. Based on anthropometric measurements of handball players presenting different levels of sporting advancement Massuca and Fragoso (2011) [10] performer that the players presenting the highest sports level were taller, heavier, and had less body fat and a larger lean mass. Gorostiaga et al. (2005) [8] compared the body structure of an elite and amateur team of handball players. It was noticed that the elite handball players presented higher values of body weight and free fat mass.

Besides evaluation of competitor’s basic somatic parameters [8,10,11], motor skills [6,12], sports seniority [9,13] it seems essential to deal with evaluation of their psychomotor abilities. Ability to predict the movement of the opponent and the ball, selective attention, choosing the response, speed of perception and a high level of sensory and motor fitness are the elements of a sports competition that help a competitor win the game [14,15]. Thanks to such skills as the ability to acquire visual information about an approaching object (ball), a high level of eye-hand coordination players can react to external stimuli more effectively and adapt their movements to the situation on the court [16]. In mentioned researches also were showed that perceptive abilities combined with the ability to predict movement and share attention help to reach successes in team sports [17]. Because competition in sports games takes place in environments varying for space and time an important aspect is quick flow of information, focusing, anticipation, predicting movement of the opponent and the ability to quickly move around the court under circumstances of a sports competition [15]. Thus psychomotor abilities, including reaction time can play a key role and lead to victory [9,15,18,19,20]. This thesis is confirmed by research conducted by Yüksel & Tunç [20], who evaluated the reaction time of a 45-person group of badminton players taking part in the 5th International Sport Games of Rumia. Based on the research, it was established that the reaction time of the leading teams were shorter than the teams that were classified below. It was also found that this factor, apart from the technique and tactical preparation, had influence on the victory [20].

The previous research shows that reaction time is influenced by the following factors such as: body height [11], training level, [13], dominant hand [21]. In addition, the results of research made by Wik et al. (2019) [22] showed the importance of training programs, which should be adapted to the position on which player plays and the league in which the athletes is competing. Badau et al. (2018) [23] made an assessment of the simple reaction time on visual stimuli among athletes practicing various sports disciplines with a dominant and non-dominant hand.

The main purpose of the paper was to evaluate selected psychomotor abilities of professional handball players using computer systems Test2Drive. The data of handball players were evaluated in relation to the control group consisting of non-training men. The impact of the competition class (league), position on the court, training seniority and the dominant hand on the studied psychomotor abilities were analyzed.

## 2. Materials and Methods

### 2.1. Characteristics of the Study Group

The study population consisted of 40 professional handball players (age: 24.02 ± 3.99, level of experience: 11.93 ± 4.43), while 50 non-training men (age: 22.90 ± 1.13) formed the control group. The league in which the players played (Superliga, 1st and 2nd league of the Polish men’s handball), position on the court (centre player, goalkeeper, pivot player and wing player), seniority (less than 10 years, from 10 to 14 years and over 14 years) and the dominant upper limb (left hand, right hand) were taken into account in the analysis of the players’ psychomotor abilities. Characteristic of the handball group with analyzed sub-groups was presented in Table 1. European Handball Federation also unified naming of each position on the court: Goalkeeper (GK), Central Player (CP), Pivot Player (PP) and Wing Player (WP). Goalkeeper takes part in a game, when he sparks the fast attacks, co-operates with defense and prevents the opposing team’s fast attacks. Pivot player performs the shot facing backwards, forwards, right or left sideways the goal. From the position of a pivot player the jump shot while falling can be executed forwards to or sideways from the goal. Wing Player, if the opposing team uses the zone defense, the feint is widely used by wing players. Centre Player is a creative handball player who directs play in both: defense and attack. Also known as the “playmaker” and sets up the tactics and the players in shooting positions. Left and right backs are usually the highest players in handball team.

The study was carried out in 2017. The scope and project research were assessed by the Bioethics Committee of the University of Rzeszow, which declared no contraindications or ethical violations (Resolution No. 02/05/2016 of 2 February 2016).

### 2.2. Measurement of Psychomotor Abilities

**Figure 1:**
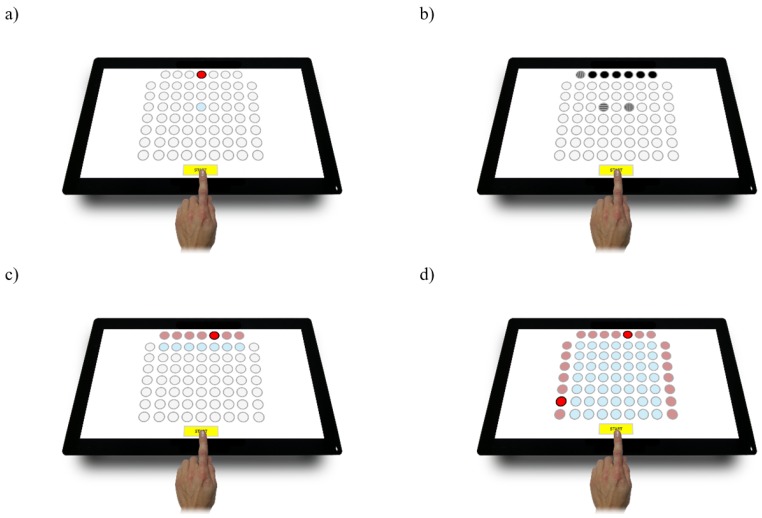
Reaction panel of the Test2Drive system; (**a**) SIRT, (**b**) CHORT, (**c**) HECOR, (**d**) SPANT.

Psychometric computer tests in the Test2Drive system (ALTA, Siemianowice Slaskie, Poland) were the study method (Figure 1). The Test2Drive system was subjected to various validation and standardization studies in the years 2013–2016. The review and characteristics of the conducted tests confirming the theoretical validity of the tests used in the system are described in the work of Tarnowski et al. [24]. The system fulfills all requirements of Regulation of the Minister of Health of 8 July 2014 regarding psychological tests of psychomotor abilities. The following four tests were used to measure the indicators of psychomotor abilities: simple reaction time test (SIRT), choice reaction time test (CHORT), hand-eye coordination test (HECOR) and spatial anticipation test (SPANT). The reaction times (RT) and movement times (MT) were the indicators in all tests, while the percentage of correct responses was additionally analysed in the CHORT and SPANT tests.

Every test was performed in a standing position facilitating access to the screen area. The parameters of the tests are presented in Table 2. The screen was in a horizontal position during the tests. At the beginning, each study participant received detailed instructions on each of the tests. Following the instructions, the exercise stage took place during which the study participants could learn the method of stimuli presentation and giving responses. The exercise stage was followed by the proper testing stage. The study participants had to react as quickly as possible to the stimuli in all tests. The study consisted of the following tests:

- SIRT—evaluation of reaction speed and its stability. The stimuli signalling field changed its colour in the right moments of time. The reaction to the stimuli involved moving the finger from the START field to the reaction time field marked in blue.
- CHORT—evaluation of the speed and adequacy of reaction in a complex situation. Horizontal (stimuli) benchmarks and vertical stimuli which require a reaction, and a slant benchmark (neutral stimuli) that does not require reaction were displayed in the top signalling row. The response to the stimuli involved moving one’s finger from the START field to one of the two reaction fields (vertical or horizontal stimulus field). During the neutral stimulus, the finger was kept on the START field.
- HECOR—evaluation of eye–hand coordination. The test required careful observation of the board and a quick reaction to the displayed red signalling field. The test participant was supposed to move his finger from the START field to the blue reaction field and return the finger to the START field.
- SPANT—evaluation of eye-hand coordination using complex spatial information. On the top, left and right of the test board there were signalling fields two of which (on in the row and one in a column) turned red simultaneously. In response to the stimulus the test participant was supposed to indicate with his finger the field on the crossing of the lit row and column, and put the finger back to the START field.

### 2.3. Statistical Methods

Basic statistical measures i.e., arithmetic mean, standard deviation and coefficient of variability were used in the study. In order to identify the significance of differences between the groups the Mann-Whitney-Wilcoxon test was used. The effect size was calculated using formula [25]:

(1) $$r=\frac{Z}{\sqrt{N}}$$

where: *Z*—standardized value for the U-value, *r*—correlation coefficient where *r* assumes the value ranging from −1.00 to 1.00, *N*—the total number of observations on which Z is based. The analysis was performed with the GNU R software [26].

## 3. Results

### 3.1. Handball Players vs. Control Group

The results obtained for handball players were compared with the results for the control group (Table 3). Analysing the SIRT results it can be observed that handball players are characterised by quicker SIRT than the control group. The RT of the players amounted to 331.0 ms, while the result for the control group was 348.3 ms (d = 17.3 ms). A similar situation can be observed in the MT analysis. Greater variability was noted in the control group both for RT and MT.

Table 2 also presents CHORT characteristics. The performed analysis showed that handball players had better RT and MT results. Statistically significant differences were observed between the studied groups (*p* < 0.05) for the studied parameters. HECOR parameters were analysed on the following stage (Table 3). Statistically significant differences were noted for the majority of HECOR variables between the study groups (*p* < 0.05). Handball players obtained better results than the non-training group. Lower diversification inside the groups was observed among handball players for the variables. The research also showed that the RT and MT measured during SPANT among the players was significantly lower than in the control group. Moreover, it can be observed that handball players gave more correct responses in the SPANT (93.1%) than the control group (88.1%). The obtained results revealed statistical significance.

### 3.2. Reaction and Movement Time of Handball Players

**Figure 2:**
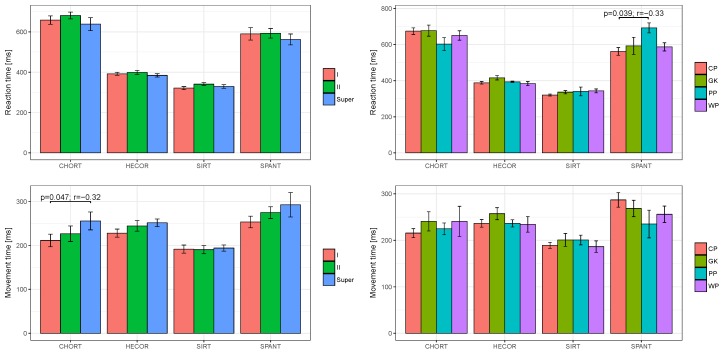
League of games; I—1st league, II—2nd league, Super—Superliga; Player’s position on the court; CP—centre player, GK—goalkeeper, PP—pivot player, WP—wing player.

Figure 2 presents results of psychomotor tests (RT and MT) depending on the league. Considering the SIRT variables it can be observed that 1st league players presented the best RT (321.0 ms), followed by Superliga players (329.2 ms), while the 2nd league players had the poorest results (340.6 ms). The situation in the MT variable analysis in the SIRT test is different. The 2nd league players demonstrated the best MT (190.4 ms) as compared to the 1st league (191.7 ms) and Superliga players (194.1 ms). The fastest RT in CHORT characterised the players who played on top of the league (638.2 ms). At the same time Superliga players had the poorest MT in CHORT (255.8 ms). Additionally, statistically significant differences (*p* < 0.05) were observed among the 1st league and Superliga players for the MT obtained in the CHORT test. The tests also revealed that Superliga handball players had the shortest RT in HECOR (384.3 ms). Analysing the second HECOR variable it can be observed that Superliga handball players had the worst MT (251.4 ms) as compared to other players. The best MT was recorded for the 1st league players (228.0 ms). SPANT was the last analysed test. The tests revealed that Superliga players had the best RT (562.2 ms) and the longest MT (292.6 ms). The 1st league players demonstrated the highest MT level in SPANT (253.3 ms).

On the following stage of the study, the RT and MT were analysed depending to the player’s position on the court (Figure 2). The analysis revealed that CPs had the fastest RT in SIRT (320.2 ms), followed by GKs (336.2 ms) and PPs (340.3 ms), while the longest RT was observed for WPs (343.4 ms). The case for MT is different. WPs recorded the shortest MT in SIRT (186.4 ms). For other players the MT results were on a similar level. An analysis of CHORT shows that PPs had the best RT (602.7 ms), while WPs, GKs and CPs achieved much poorer results. Analysing the MT in CHORT it can be observed that CPs demonstrate the best MT (215.5 ms) as compared to other players. The results obtained by GKs and WPs were on a similar level, with a small advantage in favour for GKs. The analysis also revealed that mean RT values measured during HECOR among CPs, PPs and WPs were on a similar level with a slight advantage in favour of WPs (383.7 ms). GKs had the poorest RT in the test (415.6 ms). Analysing the last psychomotor test—SPANT—it can be observed that the results obtained by GKs, WPs and CPs were on similar levels. The best RT in the groups was achieved by CPs (561.8 ms), while PPs showed the poorest RT in SPANT (692.3 ms). At the same time, PPs reached the best MT in SPANT (235.0 ms).

**Figure 3:**
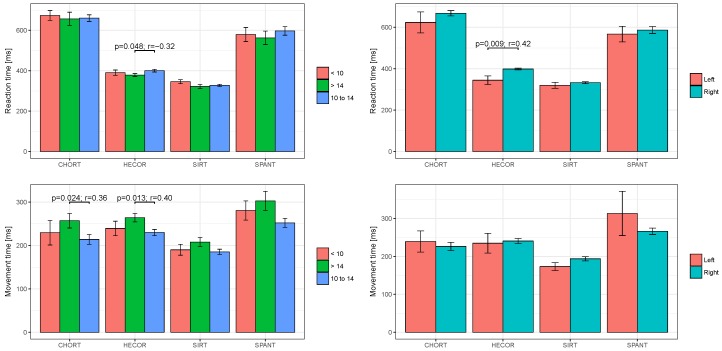
Player’s training seniority; <10—less than 10 years, 10 to 14—seniority from 10 to 14 years, >14—over 14 years; Dominant hand; Left—dominant left hand, Right—dominant right hand.

A further analysis presents the RT and MT variables depending on the player’s seniority (Figure 3). As regards SIRT it can be observed that players with the longest seniority (>14 years) were characterised by the shortest RT (321.6 ms), while players with seniority below 10 years rendered the longest RT (345.9 ms). For the MT measured in SIRT one can see that the scores of players with the shortest seniority and seniority between 10 and 14 years are on similar levels. The RT results in CHORT for each research group take similar values, with a slight advantage in favour of players with seniority over 14 years (656.8 ms). Players in this group were also characterised by the longest MT in CHORT (257.0 ms). The MT analysis in HECOR disclosed that players with seniority between 10 and 14 years achieved the best results (229.8 ms), followed by players with seniority of less than 10 years (239.3 ms) and finally the players with the longest seniority (264.0 ms). SPANT was the last analysed test. Similarly to the RT measured in HECOR, it was discovered that the players with the longest seniority achieved the best RT results in SPANT (563.0 ms).

Studies of psychomotor abilities of handball players included the aspect of the players’ dominant hand (Figure 3). Based on the obtained results, statistically significant differences (*p* < 0.05) were demonstrated between reaction times (RT) in the hand-eye coordination test (HECOR). In most of the tests, left-handed players achieved better mean values characterising the reaction time, apart from two complex tests, i.e., MT CHORT and MT SPANT, in which the right-handed players reached slightly shorter reaction time.

## 4. Discussion

Evaluation of the coordination level of psychomotor abilities in handball players was the subject matter of the study. The evaluation was made in the aspect of differences between training and non-training participants, as well as within the group of handball players with regard to the league (Superliga, 1st league, 2nd league), position on the court (centre player, goalkeeper, pivot player, wing player), sports seniority (less than 10 years, from 10 to 14 years, over 14 years) and the dominant hand (right, left).

The literature highlights a positive impact of physical exercises on the level of psychomotor abilities [13,17,27,28]. Nakamoto and Mori observed that basketball players and baseball players demonstrated faster reaction times than non-training people. Similar regularities were observed among martial art athletes [28]. Having examined 20 teenagers who did Taekwondo regularly Fong et al. discovered that people who did this sports discipline had shorter reaction time than their non-training counterparts. According to Kashihara and Nakahara on the initial stage of physical exercises, the reaction time of the study participants (men) improved [27]. It was also observed that the level of eye-hand coordination depended on the specific nature of a sports discipline. According to Grigore et al., in disciplines in which there is a direct contact with an opponent (handball, basketball, karate), the level of eye-hand coordination is much higher than in players/ sportspeople doing contactless sports (gymnastics, dance, sprint and swimming) [12]. The analyses performed in the previous chapter showed that in all four tests studying psychomotor abilities, handball players presented a much higher level of the abilities than non-training people, and the differences observed between the groups were statistically significant. In SIRT (RT i MT) handball players achieved shorter times (respectively 331.0 ms and 191.7 ms) than non-training participants of the study (respectively 348.3 ms and 264.3 ms).The study revealed also that handball players present a higher level of eye-hand coordination as compared to the study participants who did not play any sports. In HECOR test the difference in the RT and MT values between the group of handball players and the control group was 171 ms and 82.1 ms, respectively. For the majority of the studied parameters, the training group was characterised by greater cohesion (lower diversity within the group). Then a conclusion can be drawn that physical exercises have a positive impact on the level of the abovementioned abilities.

The results of studies carried out with regard to the game league show that Superliga players have the highest level of psychomotor abilities. They achieved the shortest reaction times in all tests (except SIRT). The results in this group were 638.2 ms for CHORT, 384.3 ms for HECOR and 562.2 ms for SPANT test. It was also observed that Superliga players had the poorest MT in each of the analysed tests. The 1st league players reached the best MT (except SIRT). Williams and Walmslay came to similar conclusions [29]. According to their studies, better reaction time results were noted for professional fencers, while for the MT the group had poorer results than the beginners [29]. The results are partly confirmed by studies carried out in a group of professional tennis players. Players playing on the top of the league had shorter reaction times than amateur players (Hughes et al. 1993). Gutierrez-Davila et al. [30] drew similar conclusions studying a group of fencers. Their analysis revealed that choice reaction time was not a factor differentiating the elite group from medium-level fencers [30].

The literature contains many papers investigating the relationship between position on the court and movement abilities [31,32,33,34]. Krüger et al. [31] research showed that competitors who played on the wings and back position present the best results for jumping ability. Similar phenomenon was observed during the analysis of the throwing velocity. Backs and wings handball players performed best. Another study involved evaluation of anthropometric parameters considering the player’s position [35,36]. The research conducted on rugby players group indicated that props were taller, heavier and had greater skinfold thickness than all other positions [35]. Gil et al. [36] made anthropometric and physiological characteristic of young soccer players according to their playing position. The results showed that goalkeepers were the tallest and the heaviest players. A subsequent stage of the analysis included evaluation of the level of psychomotor abilities of handball players among CPs, GKs, PPs and WPs. The analysis revealed that centre players achieved the best RT in SIRT and SPANT, pivot players were characterised by the best eye-hand coordination, while wing players recorded the best movement times in SIRT and HECOR.

Age is a factor which greatly affects reaction time. According to the studies by Luchies et al. [37], reaction time extends with the age of the study participants. The phenomenon was also observed by Redfern et al. [38]. Their observations revealed that reaction time to acoustic and visual signals, measured during the platform perturbations was longer in older participants. The results presented by Araki and Choshi [39], VaezMousavi et al. [40] and Richards et al. [41] show that the reaction time is shorter at medium level of excitement. It becomes worse when the person is too relaxed or too tense. It depends on the training seniority. The greater the player’s experience, the lower the excitement and muscle tension, and hence a shorter reaction time. The thesis is partly confirmed by this study. The performed analysis revealed that the shortest RT among handball players was recorded in the group of players with the longest seniority (>14 years). The phenomenon was observed in all tests, simple and choice reaction time, hand-eye coordination and spacial anticipation. The data analysis for the player’s seniority showed that as regards MT, the highest level of psychomotor abilities characterised players doing sports between 10 and 14 years. The worst MT and RT results for all studied psychomotor abilities were observed for players with the shortest seniority (<10 years). A conclusion can be made that regular professional training will contribute to improvement of psychomotor abilities.

Considering the issues related to the level of psychomotor abilities one shall take lateralisation into account. Evaluation of functional asymmetry of the right and left side of the human body was studied e.g., by Bryden [42], who examined only right-handed people and observed that the difficulty of the task to be performed did not significantly affect the reaction time between the right and left hand. Lateralisation can be observed in sports. Studies carried out by Al. Awamleh et al. revealed no significant difference between right- and left-handed players in the reaction time. The results do not correlate with the results obtained by Dane and Erzurumluoglu [21] who did not observe significant differences in the reaction times of the right- and left-handed handball players in the right-hand test. The researchers discovered that left-handed players were much quicker than the right-handed ones in the tests using left hand. Holtzen [43] reached similar conclusions in his study on a group of professional tennis players. According to Holtzen, left-handed players do eye-hand tasks better than right-handed players. The authors’ own study partly confirms the thesis. In the tests of psychomotor abilities, left-handed players demonstrated shorter mean values characteristic of the reaction time. Movement times in complex tests (CHORT and SPANT) were an exception because right-handed players achieved better results.

The presented paper has potential limitations. Study limitations are related to the number of handball players. In the group of 40 players there is only three of pivot players and four left handed players. The conclusions being drawn about those subgroups may be potentially not precise.

## 5. Conclusions

The evaluation of psychomotor abilities using i.e., Test2Drive can help handball coaches in appropriate selection of individual abilities of players. The increasing tempo of handball game is related to the constant improvement of the technical skills of handball players, so the time of contact with the ball is shorter. Professional handball training using psychomotor abilities should be recommended for coaches to improve the tempo of game, anticipation and decisiveness in the one-on-one game play. The present findings shows that the key point is using during training exercises connected with psychomotor abilities a special reaction time. Studies also showed that reaction and movement time is related to the level of handball game. This fact shows that it is crucial to develop and improve the psychomotor abilities in handball training process.

The following conclusions were formulated based on the analysis of the data:

- Handball players demonstrate a higher level of the analysed psychomotor abilities as compared to the control group (non-training people). The differences in time indicators among groups in all tests are statistically significant (*p* < 0.05).
- An analysis of psychomotor abilities of handball players with regard to the league revealed that in the majority of tests the Superliga players had a shorter reaction time than players in lower leagues.
- Both, position on the court and training seniority have got an impact on indicators of psychomotor abilities.
- The factor which differentiates the level of the studied skills is the dominant hand: left-handed players were characterised by shorter reaction times.
- The data obtained may be helpful in the development and optimization of training systems depending on the position on the court.

## Figures and Tables

**Table 1:** Characetristic of participants.

Groups	*N*	%
**Total Handball players**	**40**	**100.0**
**League of games**		
I—1st league	14	35.0
II—2nd league	16	40.0
Super league	10	25.0
**Player’s position onthe court**		
CP—centre player	18	45.0
GK—goalkeeper	8	20.0
PP—pivot player	3	7.5
WP—wing player	11	27.5
**Player ’s training seniority**		
less than 10 years	10	25.0
from 10 to 14 years	21	52.5
over 14 year	9	22.5
**Dominant hand**		
left hand	4	10.0
right hand	36	90.0
**Control group**	**50**	**100.0**

**Table 2:** Description of psychomotor tests in the Test2Drive system.

Test Type	SIRT	CHORT	HECOR	SPANT
Number of stimuli	20	24	20	20
Stimuli exposure time	3 s	3 s	3 s	3 s
Intervals between stimuli	1 s, 1.5 s or 2 s	1 s, 1.5 s or 2 s	1 s, 1.5 s or 2 s	1 s, 1.5 s or 2 s
Test time	3 min	3 min	3 min	3 min
Indicators	RT, MT	RT, MT, % of c.r	RT, MT	RT, MT, % of c.r.

RT—reaction time, MT—movement time , c.r.—correct responses.

**Table 3:** Numeral characteristics of psychomotor abilities of handball players vs control group.

Variable	H (*N* = 40)					C (*N* = 50)					d	*p*	*r*
	$\bar{x}$	*min*	*max*	SD	*V*	$\bar{x}$	*min*	*max*	SD	*V*	(H-C)		
**Simple Reaction Time (SIRT)**													
RT [ms]	331	282	401	29	8.7	348.3	278	500	41.2	11.8	−17.3	0.037 *	−0.22
MT [ms]	191.7	134	278	32.3	16.8	264.3	130	448	65	24.6	−72.6	0.000 *	−0.58
**Choice Reaction Time (CHORT) Test**													
RT [ms]	663.1	531	820	78.6	11.9	696.5	562	864	72.8	10.5	−33.4	0.040 *	−0.22
MT [ms]	227.9	146	461	63.3	27.8	292	156	410	65.1	22.3	−64.2	0.000 *	−0.49
c.r. [%]	93.3	62	100	7.3	7.9	93.8	62	100	9	9.6	−0.5	0.247	−0.13
**Hand-Eye Coordination Test (HECOR)**													
RT [ms]	393	289	461	32.6	8.3	410	352	516	34.9	8.5	−17.1	0.036 *	−0.22
MT [ms]	240.2	170	370	39.4	16.4	322.2	154	465	72	22.3	−82.1	0.000 *	−0.60
**Spatial Anticipation Test (SPANT)**													
RT [ms]	584.7	430	838	97.8	16.7	646	461	920	116	18	−61.3	0.014 *	−0.26
MT [ms]	271.1	162	469	60.2	22.2	321.5	201	443	60.9	18.9	−50.4	0.000 *	−0.38
c.r. [%]	93.1	75	100	6.3	6.8	88.1	20	100	17.8	20.2	5	0.886	0.02

H—handball players group, C—control group, c.r.—correct responses; *x*—mean value, *min*—minimum value, *max*—maximum value, SD—standard deviation, *V*—coefficient of variation, *p*—probability of testing, *r*—effect size for the the Mann-Whitney-Wilcoxon test. *—statistical significance.
